# *Streptococcus oralis* pulmonic valve endocarditis: a case report and review of the literature

**DOI:** 10.1186/s13256-023-03835-y

**Published:** 2023-04-03

**Authors:** Sarah B. Nahhal, Patrick Sarkis, Aline El Zakhem, Mohammad Samir Arnaout, Abdul Rahman Bizri

**Affiliations:** 1 Infectious Diseases Division, American University of Beirut Medical Center, Beirut, Lebanon; 2 Cardiology Division, American University of Beirut Medical Center, Beirut, Lebanon

**Keywords:** Case report, Infective endocarditis, Pulmonic valve, *Streptococcus oralis*

## Abstract

**Background:**

Several factors increase the risk of right-sided endocarditis. The tricuspid valve is usually involved in right-sided endocarditis cases. Infective endocarditis of the pulmonic valve is rare, and few cases of pulmonic valve endocarditis were reported previously.

**Case presentation:**

Here we describe a case of a 81-year-old Middle Eastern male patient, admitted to our hospital three times in a period of 2 months for fever and cough. He had *Streptococcus oralis* bacteremia with vegetation that was on the pulmonic valve. We diagnosed him with pulmonic valve endocarditis, and he was treated successfully with intravenous antibiotics.

**Conclusion:**

It is important to keep high suspicion for isolated pulmonic valve endocarditis in patients with respiratory symptoms. Adequate dental care is important in patients with risk factors for infective endocarditis.

## Introduction

Right-sided endocarditis is less common than left-sided endocarditis, and it constitutes around 10% of all infective endocarditis cases [1]. Several factors increase the risk of right-sided endocarditis: intravenous drug use (IVDU), degenerative valve disease, prosthetic valves, indwelling catheters and implanted cardiac devices, diabetes mellitus (DM), immunosuppression, and congenital heart disease [2]. Right-sided endocarditis cases typically involve the tricuspid valve. Infective endocarditis of the pulmonic valve is rare. Few cases of pulmonic valve endocarditis have been reported until now [3].

In this article, we report a case of pulmonic valve endocarditis due to *Streptococcus oralis* in a patient who had repeated admissions to our hospital over a period of 2 months for respiratory symptoms.

## Case presentation

An 81-year-old Middle Eastern male patient with unremarkable medical family history and with DM, hypertension, and multinodular goiter as past medical history was admitted to our hospital three times over a period of 2 months for fever and cough (Table 1). His complaints started with an acute onset fever reaching 39 °C and a dry cough after 3 weeks of the second Pfizer coronavirus disease 2019 (COVID-19) vaccine dose. His symptoms were initially attributed to the vaccine. Later he was started on an oral course of levofloxacin, but he did not respond. He was admitted to our medical center for further management.

**Table 1:** The timeline of the patient’s hospital admissions

	First admission	Second admission	Third admission
Symptoms	Fever and dry cough	Dry cough and chills	Fever and dry cough
Antibiotics before presentation	Levofloxacin	Levofloxacin	None
Investigations	General blood work, CT-chest, blood cultures	General blood work, CT-chest, blood cultures	General blood work, CT-chest, blood cultures
Results	1. pulmonic infiltrates 2. Negative blood cultures	1. Ground glass opacities 2. Negative blood cultures	1. Lung nodules 2. Positive blood cultures
Diagnosis	Severe community-acquired pneumonia	Hospital-acquired pneumonia	Right-sided endocarditis
Treatment	Piperacillin-tazobactam	Cefepime	Ceftriaxone

*CT-chest* Computed tomography of the chest

During the first hospital admission, the computed tomography (CT)-chest findings of the patient were consistent with multilobar pneumonia. He received a course of piperacillin-tazobactam for 7 days and was discharged home off antibiotics after he showed marked improvement in symptoms and laboratory results. After 2 weeks of discharge from the hospital, the patient was readmitted to our medical center for dry cough and chills. There was no documented fever. He took 3 days of oral (PO) levofloxacin at home. The CT-chest showed new ground glass opacities at the right lower lobe, so he was treated with 1 week of cefepime as a case of hospital-acquired pneumonia with the recent intake of piperacillin-tazobactam.

To note that during these two admissions, he had marked improvement in his respiratory symptoms as well as his laboratory findings. For example, the procalcitonin level in the first hospital admission was 10.4 ng/ml on the first day, it decreased to 1.18 ng/ml at the end of therapy, and it was 35 ng/ml at the first day during the second hospital admission where it then decreased to 0.16 ng/ml before discharge. Blood and urine cultures were negative, and he was discharged after being afebrile for 72 hours on both occasions.

Three weeks after the second hospital admission, the patient presented back to the emergency department (ED) with a high-grade fever, chills, and dry cough. On physical examination, he was found to have diffuse rhonchi on lung auscultation, while the remaining examination was unremarkable. CT-chest showed bilateral scattered lung nodules. He was started on meropenem to treat new hospital-acquired pneumonia. Three sets of blood cultures were taken, separated by more than 12 hours, and the three sets grew multisensitive *Streptococcus oralis* in five out of six bottles.

He was started on ceftriaxone according to the culture results and susceptibilities, including the minimum inhibitory concentrations (MICs) (Table 2). The repeated cultures after 48 hour were negative.

**Table 2:** *Streptococcus oralis* ceftriaxone and penicillin MICs according to the Clinical and Laboratory Standards Institute (CLSI) criteria

	Susceptibility			
	Ceftriaxone	0.032 µg/ml	Susceptible	
	Penicillin	0.023 µg/ml	Susceptible	

Transthoracic echocardiography (TTE) was done. It showed mild aortic valve regurgitation, mild mitral valve regurgitation, and pulmonic valve regurgitation, which was considered more severe compared with an old TTE result for the patient at our medical center. With the positive blood cultures for viridans group streptococci and the new valvular disease, we had two major clinical criteria for definite infective endocarditis. The patient refused any invasive procedure, so transesophageal echocardiogram (TEE) was not done. Instead, we did a positron emission tomography (PET)–CT scan to search for any vulvar vegetation; however, it was negative.

**Fig. 1:**
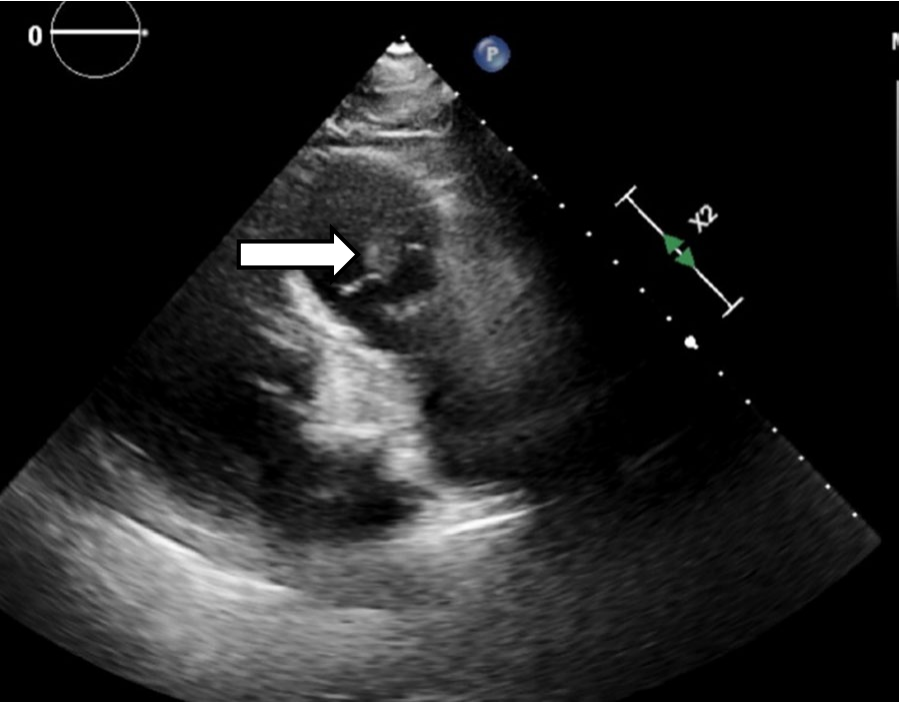
Cardiac ultrasound photo showing the pulmonic valve with vegetation (indicated by arrow)overlying the valve cusps

After ten days of receiving specific treatment with intravenous antibiotics, TTE was repeated to compare the valvular problems with the previous TTE that was done on admission and to decide if any surgical intervention was needed. It showed a mass measuring 14 × 9 mm consistent with pulmonic valve vegetation (Fig. 1).

The patient’s diagnosis was right-sided pulmonic valve endocarditis due to *Streptococcus oralis*. No surgical intervention was needed. He received treatment with a high dose of ceftriaxone for 4 weeks.

An oral panoramic image was ordered to search for any dental problem causing secondary bacteremia. It showed a dental abscess that was managed by the dentist while he was on antibiotics. Repeated TTE after 1 month of treatment showed improvement in the pulmonic valve function and persistent vegetation.

## Discussion

Infective endocarditis (IE) is when vegetation forms on the valve leaflets or cusps by the coalescence of the organisms, acute inflammation, and thrombi. Right-sided infective endocarditis is less common than that of the left side, and it occurs in the presence of risk factors such as IVDU, corrected or uncorrected congenital heart disease (CHD), and the presence of cardiac devices. Usually, the tricuspid valve is involved more than the pulmonic valve [4].

The first case of pulmonic valve vegetation was reported in 1977 in a patient who was a IVDU. [5]. Less than 2% of patients with infective endocarditis have isolated pulmonic valve endocarditis[6]. Since it is rare, the data on pulmonic valve infective endocarditis stems from case reports and few published case series [7].

Pulmonic valve (PV) IE can present as pneumonia—which was the case in our patient, pleural effusion, or pulmonic infarction caused by vegetation septic emboli [5]. PV regurgitation murmur develops late in the disease [5], and it is usually hard to detect during physical examinations since it is low-pitched and short [7].

A total of 91% of the isolated PV IE cases are diagnosed with TTE, which provides good visualization of the PV cusps [7]. This was illustrated in our case. It has a sensitivity of 30–63% and a specificity of 91–100% for PV endocarditis diagnosis [5]. Usually, all the patients with pulmonic valve endocarditis have relatively large vegetation, greater than 1 cm [7]. On the contrary, TEE may not give optimal images of the pulmonic valve owing to its anterior location, which makes it farthest from the TEE probe and limits the imaging results [7]. So, clinicians need to use TTE as the modality of diagnosis once PV endocarditis is suspected.

18F-fluorodeoxyglucose (FDG)-PET–CT is used in the diagnosis of infective endocarditis, and it showed high accuracy for the prosthetic valve endocarditis diagnosis, but there is limited data regarding native valve endocarditis patients [8]. It showed a negative result in our patient.

Some risk factors predispose the patient to pulmonic valve endocarditis, including IVDU, diabetes mellitus, CHD, and central venous catheters, but 28% of the cases have no known risk factors [5]. Our patient was diabetic.

The pathogenesis of right-sided endocarditis is explained by different hypotheses such as the unusual immunological phenomena and endothelial damage that result from the injected drugs in IVDU, which lead to vegetation formation on the valves [9].

The most common pathogens discovered causing pulmonic valve endocarditis are *Streptococcus viridans* (*S. viridans*) (among which is *S. oralis*) and *Staphylococcus aureus* [7]. From 55% to 65% of nondrug user cases are caused by *S. viridans*, whereas *Staphylococcus aureus* predominates in drug users [5].

The normal flora of the human oral cavity includes multiple groups of organisms, with the viridans streptococci group forming a significant part of it [10]. Different disease conditions are caused by the *Streptococcus viridans* group other than IE, and this includes dental caries, sepsis in neutropenic patients, and purulent infections of the oral cavity and other body sites [10].

The most common causes of infective endocarditis are streptococcal bloodstream infections [11], and the most common species were *S. sangius* (31.9%), *S. oralis* (29.8%), and *S. gordoni* (12.7%) [10]. The prevalence of *Streptococcus oralis* endocarditis among native valve disease patients is 7%, and the prevalence among patients with prosthetic valve endocarditis is 5% [11]. The mechanism of *Streptococcus oralis* endocarditis is not very well known, it is postulated that that *S. oralis* can grow more in plasma or thrombotic vegetations compared with other oral *Streptococci* species [10].

There are few published case reports about *Streptococcus viridans* pulmonic valve endocarditis (Table 3) and only one case specified *Streptococcus oralis* in which the patient presented with neck pain [12].

**Table 3:** Reported cases of *Streptococcus viridans* pulmonic valve endocarditis

Age	Gender	Organism	Valve	Year	Presentation	References
24	Male	*Streptococcus viridans*	Pulmonic	2000–2014	Not mentioned	[7]
48	Female	*Streptococcus viridans*	Pulmonic	2000–2014	Not mentioned	[7]
52	Male	*Streptococcus oralis*	Pulmonic	2015	Neck pain and fever	[12]
41	Female	*Streptococcus mutans*	Pulmonic	2018	Pulmonic artery embolus and lung nodules	[13]
47	Male	*Streptococcus mitis*	Pulmonic	2019	Flu like symptoms	[6]
47	Female	*Streptococcus viridans*	4 valves	2019	Worsening non pruritic rash	[14]
74	Female	*Streptococcus mitis*	Pulmonic	2019	Respiratory symptoms and pneumonia	[15]
81	Male	*Streptococcus oralis*	Pulmonic	2021	Respiratory symptoms and pneumonia	Our patient

Bactericidal antibiotics are usually used for the treatment of infective endocarditis in an attempt to eradicate the infection over a prolonged period, usually 4 weeks or more. Penicillin minimum inhibitory concentration data are used for the treatment of streptococcal IE due to viridans group streptococci [2]. The concentration must be ≤ 0.12 μg/ml [13]. And, in patients infected with strains of streptococci that are susceptible to penicillin, monotherapy with parenteral penicillin or ceftriaxone is highly effective [2].

Our patient had *Streptococcus oralis* bacteremia secondary to a dental abscess. He received 4 weeks of intravenous ceftriaxone. The negative blood cultures on the first two hospital admissions were because he received oral levofloxacin at home before presenting to the ED. The partial improvement on intravenous antibiotics in the first two hospital admissions increases the likelihood that the diagnosis on initial presentation was infective endocarditis, and it presented with respiratory symptoms. Since IE requires prolonged therapy, our patient had a recurrence of symptoms after stopping intravenous antibiotics in the first two hospital admissions.

## Conclusion

This case highlights the importance of clinical suspicion of isolated pulmonic valve endocarditis in patients presenting with recurrent respiratory symptoms. Patients may be initially misdiagnosed as cases of pneumonia, such as in our case. This might delay proper therapy and predisposes patients to complications. Moreover, our case emphasizes the importance of adequate dental care in patients with risk factors for IE.

## Data Availability

The datasets during and/or analyzed during the current study are available from the corresponding author on reasonable request.

## Abbreviations

IVDU: Intravenous drug use
DM: Diabetes mellitus
ED: Emergency department
CT: Computed tomography
PO: Orally
MIC: Minimum inhibitory concentration
TTE: Transthoracic echocardiography
PET-CT: Positron emission tomography–computed tomography
IE: Infective endocarditis
IVDU: Intravenous drug use
CHD: Congenital heart disease
PV: Pulmonic valve
